# Case of Traumatic Tetanus in the Horse, Successfully Treated by the Administration of Tobacco

**Published:** 1825-09

**Authors:** P. Egan

**Affiliations:** Assistant Surgeon 12th Royal Lancers.


ART. IV.—*Case of Traumatic Tetanus in the Horse, successfully treated by the Administration of Tobacco.* By P. EGAN, A.B. &c. Assistant Surgeon 12th Royal Lancers.

TRAUMATIC tetanus may be defined a peculiar lesion or derangement of the cerebro-spinal nervous system, produced by wounds, contusions, or local irritation exciting inflammation in a nerve of the injured part, which inflammation, being transmitted to the medulla spinalis, tuber annulare, or medulla oblongata, generates that disease of the muscular system termed tetanus.

Tetanus may not be deemed of very unfrequent occurrence in regiments of cavalry in time of peace, yet in civil life may be supposed rare: in both, however, certainly too frequent, if an opinion be formed from the obstinate nature of the disease, and its fatal character.

I must consider this disease to be equally fatal in its termination in the horse as in the human species; the subject of this case being the only one of acute tetanus, accompanied with complete trismus in the former, and that treated by my old and respected friend, Mr. O'Beirne, of Dublin, in 1821, of the latter, whose successful termination I have witnessed, notwithstanding I have seen four cases of tetanus* in the human species previous to my entrance into the service, as well as numerous instances of this disease during the Spanish Peninsular campaigns, in the British and the French line of hospitals, established from Talavera, Madrid, &c. to Bayonne inclusive.

I now beg leave to describe the symptoms of tetanus in the horse, as I have happened to observe them in ten instances† which came under my notice in the course of sixteen years, which I have the honour to serve in his Majesty's cavalry.

---

* One occasioned by a wound of the scalp, treated by the late Mr. Richard Dease, of Dublin.

† One of these, idiopathic tetanus from exposure to cold and inclement weather, terminated fatally, twenty-four hours after its accession.

*Symptoms of tetanus in the horse.*—Rigidity, permanent fixation, and involuntary contraction of nearly all the voluntary muscles, especially those of the throat, jaws, neck, and abdomen; tail tremulous or shaking violently, and in a horizontal position; ears generally erect, and pricked forwards; nose thrust forwards, nostrils considerably dilated; eyes, at the commencement of the disease, wild and fiery. The animal at this period exhibits a highly interesting, eager, animated countenance. These symptoms become gradually more aggravated; difficult mastication, and subsequently difficult deglutition, is observed. The jaws at length become nearly or altogether permanently closed; the eyes now are occasionally contorted; the retractor muscle of this organ (which, with the exception of the monkey tribe of animals, is peculiar to quadrupeds,) becoming affected with spasm; the membrana nictitans is consequently pushed forwards, and covers one half of the globe of each eye, which are often, during the height of the paroxysm, drawn back into the bottom of their orbits. The legs are placed at a considerable distance from each other; the joints become stiff and rigid; sluggishness, or an inaptitude to locomotion, is observed; the abdominal muscles are drawn up; the fæces are obstinately retained, chiefly from inability to expel them; great difficulty in voiding the urine is noticed; cold perspirations frequently supervene; the patient is very much disturbed and alarmed at the least possible noise.

The appetite is not in the least impaired; and the fruitless, tantalising efforts to indulge it excite commiseration from the most indifferent spectator.

The pulse is scarcely affected, except during the paroxysms or immediately after. Locomotion at these periods, or when disturbed or alarmed, it becomes quick, hurried, and irregular. Respiration, though not much affected at the commencement, at length becomes laborious.

The paroxysms become frequent and violent, finally without any remission; and the animal, being quite exhausted from inanition and the effects of the disease, at length falls to the ground, and dies convulsed.

I have only witnessed the dissection of a few of those horses who fell a victim to this dreadful malady in the regiment; but my friend, Mr. Castley, our veterinary surgeon, assures me that he has often found the theca of the medulla spinalis discolorated, as well as its covering derived from the pia mater, both exhibiting unequivocal marks of inflammation. I can also testify that the liver, and occasionally the lungs, have been often found more or less diseased.

The subject of the case about to be described was purchased by me for my own use, at the age of four years and a half, in poor condition, having recently recovered from the strangles. At that period, and at this moment, his skin was partially covered with a crop of warts, particularly on the abdomen and legs. He is of half-blood, of a light bay colour, courageous and spirited, but by no means irritable or sulky in his disposition. He was six months from pasture, in good, but not hard condition, on the 10th of April, 1824, being at that period five years old.

On the morning of the 10th of April, 1824, a rough rider rode the horse over a small, but awkward leap (a stone wall) off the high road. In leaping back, the animal lacerated and tore off a large wart, situated on the centre of the abdomen, near the umbilicus; at the same time the near stifle (patella) joint of the hind leg was contused, and a portion of the skin abraded, for about an inch and a half longitudinally, and half an inch in breadth. The appearance of this wound was of very trifling import, and the beast, although somewhat stiff in his action immediately after the accident, did not walk lame. A purgative ball was administered that night, to obviate inflammation. In three days after, the horse was as sound, and apparently in as good health, as before the accident occurred. He was exercised daily after this as usual, until the 28th of April, eighteen days after the accident took place.

28th of April, 1824.—This evening, at eight o'clock, I happened to visit my stable, and remarked that the animal, although in good condition, appeared as if he had had no drink for twenty-four hours, his abdomen being very much tucked up: he had his usual allowance of water four hours previously. Before I left the stable, I thought I perceived his tail shake a little for a moment: he, however, ate the greater part of his corn then, and finished it before morning.

29th April.—This morning, at six o'clock, my servant reported to me, that my bay horse appeared unwell; that I could not ride him at the review (King's birth-day*); that he masticated his food with some difficulty, and that his tail shook very much. I immediately expressed my fears that he was about to be attacked with tetanus, and wrote over directly to Cork to our veterinary surgeon, if possible, to come and see the animal, or, at all events, direct me what to do. In the mean time I sent for a respectable veterinary surgeon, practising in the town (Limerick) where I was quartered at this period. Before this gentleman arrived at the barracks, I had three quarts of blood taken from the animal.

At eight o'clock A.M. I saw the horse, and with regret perceived my apprehensions were but too well founded, that tetanic

* Kept this year (1824) on the 29th, instead of 23d April.

symptoms were fast approaching: some, indeed, were actually present,—viz. partial trismus, rigidity of the muscles generally, tail tremulous, and occasionally shaking violently.

About one P.M. Mr. Deacon, a respectable veterinarian, in good practice at Limerick, visited the horse. He immediately pronounced his disease to be tetanus: he ordered three quarts more of blood to be extracted, and vainly endeavoured to administer a liquid purgative by the mouth. The horse was then rubbed with the unguentum lyttæ, along the entire vertebral column to the tail; the muscles of the throat and jaws, and the left stifle joint (the part originally contused), were also blistered. Two large purgative enemata were administered: these last were immediately rejected.

At half-past one P.M. a discharge of artillery, and a feu de joie, in honour of his Majesty's birth, taking place, although the horse was well accustomed to fire, he was seized with a complete paroxysm of acute tetanus. At three P.M. complete trismus set in. Another common purgative clyster was given, without any discharge of fæcal matter. The animal was then placed loose in a cool stable for the night.

30th April.—The blister excited copious prespiration, and has acted severely as a vesicatory. Symptoms of tetanus rather less acute than yesterday. He has contrived, by repeated efforts at suction, to take in and swallow about a pint of white-wash (oatmeal water) within the last twenty-four hours, the only sustenance he has had for this period. On the arrival of Mr. Deacon this day, this practitioner proposed the cold affusion, to which I gave a reluctant consent, having on a former similar occasion conceived it not to be productive of any good effect. This operation was performed by pouring two large buckets of cold water from the stable-loft suddenly on the spinal column; having four men ready to rub and dry the animal as quick as possible afterwards. This cold douche aggravated all the symptoms, rendered the paroxysms more intense and constant; the symptoms already detailed in the description being present, with the exception of the near eye only being occasionally drawn into the bottom of the orbit, and the globe being only partially covered by the (haws) membrana nictitans; the ears also were not pricked forward.—Common purgative enemata were administered ineffectually.

1st of May.—Tetanic symptoms acute, but not so constant as yesterday. Mr. Deacon appears to entertain no hopes of recovery. Mr. Castley, our veterinary surgeon, writes that he cannot possibly come to Limerick, being obliged to go to a fair to purchase horses for the regiment. Agreeably to this gentleman's desire, I have dissolved half an ounce of the extract of opium in four quarts of water as a drink, but the animal will not taste this liquid. He has taken in only about half a pint of white-wash this day.—Purgative enemata were administered ineffectually. Half an ounce of crude opium was placed as a suppository in the rectum. The animal was turned out all night in the open air into a forage-yard: the night was dark, rather wet, and cold.

2d May.—Tetanic paroxysms not so acute, but more constant than yesterday, the remissions being less frequent; trismus more complete, if possible, than hitherto. Has not taken any nourishment this day. His bowels have not been excited for four days, notwithstanding cathartic enemata are administered daily. The opium suppository has been removed from the rectum, and discontinued.

3d May.—Mr. Deacon considers this case hopeless. This circumstance, as well as having witnessed the successful termination of this disease, by the administration of tobacco enemata to a boy aged thirteen, by my friend Dr. O'Beirne, of Dublin, three years since, when quartered in that city, and the very scientific view that gentleman entertained of the disease in the publication* of his successful case, which he was so good as to present me with, as well as his case being the only successful termination I ever witnessed, determined me (the animal being my own property) to administer the same remedy, and give it a fair trial. Accordingly, an ounce of leaf-tobacco was infused in a quart of boiling water for about an hour; this, when sufficiently cool, was administered as an enema: it produced a discharge of dark fluid fæces from the rectum. I imagined that slight nausea was produced, and that the following tetanic paroxysm was not so acute as the preceding: this induced me to repeat the tobacco enema in the evening. He drank about a quart of white-wash this day.

4th May.—I procured half a pound weight of tobacco, boiled it for ten minutes in two quarts of water: this, when sufficiently cool, was given as an enema, the greater part of which was retained for ten minutes. It produced a considerable discharge of fluid fæcal matter, as well as some degree of sickness and nausea. It was administered in the acmé of the paroxysm, which terminated half an hour sooner than usual. In four hours, the same tobacco was boiled over again in two quarts of water, and given as before per anum. Had only one tetanic paroxysm this day; trismus nearly as before; he, however, contrived to swallow two quarts of white-wash.

5th May.—No tetanic paroxysm this day: he moves his jaw a little, and begins to pick at some fresh hay, but cannot swallow

---

* *A Case of Traumatic Tetanus successfully treated by Tobacco; with Observations.* By JAMES O'BEIRNE, M.D. Surgeon Extraordinary to the King, &c. &c.—Dublin, 1822.

it. He drank during this day, with great difficulty, four quarts of white-wash.—Tobacco enemata were discontinued.

6th May.—He was left quiet in the forage-yard all this day, and drank three quarts of whitewash. Tobacco enemata omitted. No discharge of fæces for forty-eight hours. In the evening, two common purgative enemata were given, without any effect.

7th May.—The animal this morning is affected with complete trismus: he keeps his head and neck in a drooping position close to the ground; he appears immovable; the skin and muscles feel so rigid and hard, that he has been compared to a wooden horse. The noise made by some drum-boys, practising the beats of the drum near the forage-yard, excited one acute tetanic paroxysm this day, of two hours' duration; the flitting of a bird, or the buzzing of a fly, brings on a slight paroxysm, and alarms him very much. A quarter of a pound of tobacco was put into two quarts of water, and boiled down to three pints of fluid: this, when sufficiently cool, was administered as an enema; it excited nausea and considerable debility. Enema was repeated in three hours, and once again at night, by infusing the same tobacco over again in boiling water: they produced the same constitutional effects as the first, as well as a considerable discharge of fluid fæcal matter. The same tobacco was then made into a suppository, and left all night high up in the rectum. Has not drank any white-wash, nor taken any food, this day.

8th May.—Deglutition of fluids in some degree restored, and he appears to be as well as on the 5th instant, when the tobacco enemata were intermitted. He has not had a paroxysm this day, and drank three pints of white-wash. A quarter of a pound of fresh tobacco was procured, and three tobacco enemata were given this day, as before, with similar results. The tobacco suppository was used at night.

9th and 10th of May.—The enemata were administered thrice daily as before, and the suppository at night. They excited considerable debility and nausea, and produced a general relaxation of the muscular system. Three quarts of fæcal fluid were discharged daily from the rectum; and, on the 10th, four large, hard, black, round scybalæ were discovered in the evacuations. He drank twelve quarts of white-wash during these three days; has had no paroxysm; and he begins to nip, and occasionally makes ineffectual efforts to swallow, a little fresh grass. On this last day (the 10th,) I had him turned into a nice grass field, at a short distance: he was an hour walking there (250 yards), and appeared very much fatigued and weakened by the exertion.

11th May.—Passed some hard, round, black fæces during the night; has drank five quarts of white-wash since yesterday. Two tobacco enemata were given; suppository omitted. No paroxysm.

12th and 13th May.—Drank eight quarts of white-wash on each of these days, and he contrives, by kneeling on one knee, to nip and swallow a small quantity of grass. Two enemata were given as before daily. Rigidity of the muscles diminishes rapidly. No paroxysm.

14th and 15th May.—Deglutition of fluids perfectly restored. He eats a tolerable quantity of grass, and moves about the field. He now moves his jaws, and the fingers can be introduced with ease into the mouth. Has had a discharge of black, soft fæcal matter spontaneously twice each day. Two tobacco enemata administered daily. No paroxysm.

16th May.—Mr. Deacon having heard that the horse was still alive, came this day to see the animal. This gentleman is much surprised at his appearance, and at the very unhoped-for success attending the remedy I ventured to employ. He thinks it very possible the animal may recover. Mr. D. contrived, with great difficulty, to administer a liquid purgative by the mouth; in three hours after, one tobacco enema was given: they produced two black, soft stools.

17th, 18th, and 19th May.—Passes his fæces naturally; eats a moderate quantity of grass, and drinks a sufficient quantity of white-wash; and the two last days he ate four pounds of mashed bran each day. Rigidity of the muscles has completely subsided; his sheath, however, and the upper part of his thigh, are much swelled: these are fomented with warm water; and the whole of the vertebral column, throat, jaws, and injured stifle-joint, which were severely blistered, have been rubbed well with soft lard. The tobacco enemata have been discontinued for the last three days.

20th May.—The horse approaches convalescence rapidly. He masticates corn well, but very slowly; deglutition perfect. He yawns frequently, and he opens his mouth on these occasions nearly as wide as ever.

30th May.—On this day, a month after the disease appeared, the animal seemed quite recovered in every respect: however, a very small portion of the membrana nictitans covered a corresponding part of the left eye, at the external canthus. This affection is still apparent.

1st June.—The squadron received orders to march thirty-five miles in two days: the animal was led twenty miles the first day, and fifteen the next, without being injured by the journey. From this day forward he regained his strength and appetite rapidly.

10th July.—The horse was in excellent condition, and shortly after was embarked for England with the regiment.

March, 1825.—He has been frequently hunted with the Brighton harriers this winter. He is now in the regiment, in good health and excellent condition, having lately sold him to a brother officer for sixty guineas.

***Brighton Barracks; April,* 1825.**

